# Identification, characterization and expression analysis of wheat RSH family genes under abiotic stress

**DOI:** 10.3389/fpls.2023.1283567

**Published:** 2023-11-27

**Authors:** Mengru Wang, Wei Hong, Youning Wang, Xiaowen Han, Wang Chen, Shuping Wang, Yingxin Zhang, Wenli Wang

**Affiliations:** ^1^ Ministry of Agriculture and Rural Affairs (MARA) Key Laboratory of Sustainable Crop Production in the Middle Reaches of the Yangtze River (Co-Construction by Ministry and Province), College of Agriculture, Yangtze University, Jingzhou, China; ^2^ Hubei Key Laboratory of Quality Control of Characteristic Fruits and Vegetables, Hubei Engineering University, Xiaogan, Hubei, China; ^3^ College of Plant Protection, North West Agriculture and Forestry (A&F), University, Yangling, Shaanxi, China

**Keywords:** RSH genes, bioinformatics, RT-qPCR, transcriptome analysis, abiotic stress

## Abstract

Guanosine pentaphosphate and guanosine tetraphosphate are collectively called (p)ppGpp (Guanosine tetraphosphate and pentaphosphate). (p)ppGpp content in plants is affected by conditions such as light, salt, pH, UV light, and environmental phytohormones. The synthesis and hydrolysis of (p)ppGpp in plants is accomplished by a class of proteins called RSH (RelA/SpoT homologs). To date, a systematic and comprehensive genome-wide analysis of the RSH gene family in wheat and its closely related species has not been conducted. In this study, 15, 14, 12, and 8 members of RSH were identified in wheat (*Triticum aestivum*), *Triticum dicoccoides*, *Triticum urartu* and *Aegilops tauschii* respectively. Based on the conserved structural domains of the RSH genes, the *TaRSHs* have been categorized into *TaRSH* and *TaCRSH*. The gene duplications in the TaRSH gene family were all identified as segmental duplications indicating that the TaRSH family plays a significant role in expansion and that segmental duplications maintain a degree of genetic stability. Through the analysis of transcriptome data and RT-qPCR experiments, it was observed that the expression levels of TaRSHs were upregulated in response to abiotic stress. This upregulation suggests that TaRSHs play a crucial role in enhancing the resilience of wheat to adverse environmental conditions during its growth and development. Their increased expression likely contributes to the acquisition of stress tolerance mechanisms in wheat. Especially under NaCl stress, the expression levels increased most significantly. The more detailed systematic analysis provided in this article will help us understand the role of *TaRSHs* and provide a reference for further research on its molecular biological functions in wheat.

## 1 Introduction

Wheat (*Triticum aestivum*) is a herbaceous plant and a cereal crop widely grown around the world. The safe and stable production of wheat is crucial for ensuring food security (Albahri et al., 2023). However, wheat quality and yield are often affected by abiotic stresses including drought, saline-alkali, and extreme temperatures during growth and development (Yin et al., 2018), As a consequence, it is of great importance for wheat genetic improvement to explore stress resistance-related genes and screen wheat germplasm resources with high resistance (Kosová et al., 2014).

When living organisms are under environmental stress, they can regulate their metabolism to slow down their metabolic activity, save resources, and resist adverse conditions (Rossnerova et al., 2020). The strict response is a kind of stress signaling system in response to nutritional deprivation (Irving et al., 2021). It has the ability to and promote cell survival under challenging environmental circumstances (Braeken et al., 2006). Guanosine tetra- and pentaphosphate, commonly known as (p)ppGpp or alarmones, are key molecules involved in the strict response. They are synthesized and hydrolyzed by proteins belonging to the RSH superfamily, which are homologues of RelA/SpoT proteins (Sinha and Winther, 2021). The strict response was first identified in bacteria and it is marked by the accumulation of (p)ppGpp molecules synthesized by RelA/SpoT (Jain et al., 2006).

For the past few years, with the evolution of genomics, RSH has been identified in *Arabidopsis thaliana* (van der Biezen et al., 2000), *Nicotiana tabacum* (Givens et al., 2004), *Oryza sativa* (Du et al., 2019), *Brassica napus* (Dąbrowska et al., 2021) and other plants. Furthermore, previous research has shown that RSH plays a vital role in plant response to biotic/abiotic stresses. The expression level of *AtRSH2* was significantly increased under salt stress (Mizusawa et al., 2008). Abscisic acid (ABA) is a plant hormone involved in signals transduction under environmental stress (Nakashima and Yamaguchi-Shinozaki, 2013), which can induce the *AtRSH2* expression (Mizusawa et al., 2008). Takahashi study has been found that mechanical damage can down-regulate the expression level of *AtRSH1* while up-regulate that of *AtRSH2* (Takahashi et al., 2004). In *O. sativa*, Du’s study found that the expression of *OsRSHs* are induced to varying degrees by abiotic stresses such as salt, low temperature, high temperature, and mechanical damage (Du et al., 2019). In *Brassica napus*, the expression of *BnRSHs* remains unaffected by salt stress. However, when exposed to PGPR bacteria, particularly *Serratia* sp., there is a notable increase in the expression of *BnRSHs* (Dąbrowska et al., 2021).

So far, there are no studies have systematically studied the RSH gene family in wheat. To conduct a comprehensive analysis of the wheat RSH gene family and elucidate the expression patterns of *TaRSHs* under abiotic stress, this study employed bioinformatics techniques to analyze the *TaRSH* genes. In the end, we successfully identified 15 *TaRSH* genes and conducted a comprehensive analysis of their phylogeny, protein characteristics, structural features, chromosomal distribution, transcriptome profiling, promoter analysis, Subcellular localization analysis, and their expression patterns under various abiotic stress conditions. This study elucidates the functional genes within the wheat RSH gene family, thereby yielding significant insights that pave the way for further in-depth exploration of their biological roles. The findings offer valuable information that can guide future research endeavors aimed at unraveling the intricate molecular mechanisms and regulatory networks associated with the *RSH* genes in wheat.

## 2 Materials and methods

### 2.1 Identification of RSH genes in *T. aestivum*

The wheat reference genome, IWGSC RefSeqv2.1, was acquired from Ensembl Plants database (http://plants.ensembl.org/index.html) through a download procedure. The Hidden Markov Model of RelA/SpoT (RSH) homologs (PF04607) was retrieved from Pfam database (http://pfam.xfam.org/) via a download process. And the RSH protein sequences were collected from relevant literature reports and other species databases, including four *Arabidopsis thaliana* RSHs (AtRSHs) (van der Biezen et al., 2000), six *Oryza sativa* RSHs (OsRSHs), four *Solanum lycopersicum* (SlRSHs) and eight *Zea mays* (ZmRSHs). The obtained protein sequences were employed as reference sequences to identify potential TaRSHs by performing BLASTp searches (e-value <1e^-5^) against the wheat protein database (Jiang et al., 2020). Then the protein sequences containing RelA/SpoT protein domain were detected through the Pfam (v35.0, http://Pfam.xfam.org) and InterProScan (v94.0, http://www.ebi.ac.uk/InterProScan). After eliminating the redundant and incompletely annotated sequences, the finally retained genes were identified as the members of wheat RSH family. Moreover, the *RSH* genes of the other nine wheat cultivars were obtained by blast in the 10+ Wheat Genome Project database (https://galaxy-web.ipk-gatersleben.de/).

### 2.2 Phylogenetic analysis of RSHs

The multiple sequence alignment of the identified RSHs were analyzed by ClustalW2 in MEGA 11 software (Polyakova et al., 2022). The phylogenetic tree containing TaRSHs, AtRSHs (van der Biezen et al., 2000), OsRSHs (Du et al., 2019), SlRSHs and ZmRSHs was constructed by neighbor-joining method (1000 bootstrap replicates) and modified by iTol online tool (Interactive tree of Life, http://ITOL.embl.de). The original name of SlRSHs and ZmRSHs are shown in **Supplement File Table S1**. TaRSHs were named according to the phylogenetic relationships and pfam conserved domains. And the Phylogenetic tree for the RSHs in Chinese spring and nine other wheat cultivars (SY Mattis, CDC Stanley, CDC Landmark, Norin 61, PI190962 (spelt wheat), LongReach Lancer, Julius and Jagger) were also constructed using the above methods.

### 2.3 Chromosomal location, collinearity, and Ka/Ks analysis of TaRSHs

The gene annotation information of *TaRSHs* was extracted from IWGSC v2.1 GFF3 file. The start and end location information about *TaRSHs* in correspondence chromosomes were used to draw the physical map via the MapInspect software (Song and Peng, 2019). The homologous gene pairs between *TaRSHs* and the reference genomes of *T. aestivum*, *T. urartu*, *T. dicoccoides*, and *Ae. Tauschii* (Nan et al., 2018) were identified by BLASTp (e-value<10^−10^, similarity>80%). The non-synonymous replacement rate (Ka), synonymous replacement rate (Ks) and Ka/Ks values among the *RSHs* gene pairs from the four species were calculated by TBtools (Chen et al., 2020). Finally, the interspecific collinearity map was drawn by TBtools, and the scatter map of Ka/Ks values was drawn by Excel. Collinearity analysis between wheat and between wheat and rice calculated with TBtools.

### 2.4 Analysis of the conserved domains and motifs of TaRSHs

The exon/intron structures of *TaRSH* genes were precisely delineated by employing TBtools, utilizing the comprehensive information from the GFF3 gene structure annotation (Chen et al., 2020). Conserved motifs within *TaRSH* genes were identified by employing the MEME (version 5.5.1, http://meme-suite.org/tools/meme). The motif sequence are shown in **Supplement File Table S2**.

The analysis was conducted with the following parameters: allowing each sequence to contain an arbitrary number of non-overlapping motifs, identifying a total of 10 distinct motifs, and considering motif widths ranging from 10 to 100 amino acids. This approach facilitated a comprehensive investigation of conserved motifs within *TaRSH* genes.The protein motif structure was mapped using TBtools. TaRSH conserved domain information was obtained from the pfam website and the results were visualized by TBtools.

### 2.5 Protein characterization and three-dimensional homology modeling of TaRSH

The protein fundamental characteristics of TaRSHs, such as protein molecular weight (MW), sequence length (Len), total hydrophilicity (GRAVY) and isoelectric point (pI), were assessed utilizing the ExPASy Server10 (https://prosite.expasy.org/PS50011). This analysis facilitated a comprehensive examination of the essential properties of the TaRSH proteins (Wilkins et al., 1999). To determine the subcellular localization of *TaRSH* genes, the Plant-mPLoc tool (http://www.csbio.sjtu.edu.cn/bioinf/Plantmulti) was employed. his computational approach enabled the prediction of the precise cellular compartments within plant cells where these TaRSH proteins are expected to be localized (Fang et al., 2020). The three-dimensional (3D) structure of TaRSHs was computationally modeled using SWISS-MODEL (https://www.swissmodel.expasy.org/).

### 2.6 *cis*-element analysis and protein-protein interaction network

In order to identify the *cis*-elements in the promoter sequence of the *TaRSH* genes, the upstream sequences (1-1500 bp) of *TaRSHs* were extracted from the wheat genome, and were uploaded to the PlantCARE (http://bioinformatics.psb.ugent.be/webtools/plantcare/html/plant) to identify *cis*-elements (Lescot et al., 2002). Then, the prediction result was displayed using the R package “pheatmap” (Hu, 2021). The model protein interaction networks of TaRSHs are predicted through STRING database (http://string-db.org) (Szklarczyk et al., 2015). The minimum interaction score required is set to a high confidence level (0.700); the maximum number of interactive displays no more than 10 on the first housing, the display is not more than 10 on the second housing. Then the visual image is exported from its website.

### 2.7 TaRSHs expression pattern analysis

Wheat transcriptome sequencing datas (PRJEB25639, PRJEB23056, PRJNA436817, SRP133837, PRJEB25640, and PRJEB25593) were downloaded from the NCBI-SRA database and were mapped to the wheat reference genome using Hisat (Ramírez-González et al., 2018). The *TaRSHs* expression level were calculated by Cufflinks (normalized as TPM value) (Trapnell et al., 2012). Expression patterns of TaRSHs under different conditions were heatmapped using Log_2_(TPM+1) values by R package pheatmap.

### 2.8 Targeting relationship prediction between miRNAs and TaRSHs

To identify miRNAs targeting to *TaRSHs*, the *TaRSHs* CDS sequences were uploaded to the psRNATarget online website (https://www.zhaolab.org/psRNA/) to determine the potential targeting relationships between *TaRSHs* and miRNAs (Dai et al., 2018). Afterwards, the results were visualized using R package “ggplot2” and “ggalluvial”.

### 2.9 Growth and stress treatments at the seedling stage of wheat

Wheat (Yangmai 20) seeds were sterilized by immersing them in a 1% sodium hypochlorite solution. Then, seeds were thoroughly rinsed with double distilled water. Subsequently, sterilized seeds were placed onto petri dishes layered with two sheets of saturated filter paper, and cultured for 2 days at a temperature of 25°C (Zhang et al., 2019). The germinated seeds were transferred to petri dishes containing a 1/4 strength Hoagland solution. After 3 days, the concentration of the Hoagland solution was increased to 1/2 strength. The growth conditions during the experiment are as follows: a 16-hour light period followed by an 8-hour dark period, a temperature was maintained at 25°C (Yang et al., 2022). At the one-leaf and one-heart stage of growth, the wheat seedlings were exposed to solutions of 150 mM NaCl, 20% PEG6000 and 100 μm ABA, while distilled water (ddH_2_O) was used as a control. Samples of leaf and root tissues were collected at specific time points, including 2, 12, 24, 48, 72 and 120 hours after the treatment initiation (Sahin et al., 2015).

### 2.10 RNA extraction and RT-qPCR analysis

With the aim of show the specific expression profile of *TaRSHs* in wheat tissues and during development, RT-qPCR was performed to detect the expression level of *TaRSHs*. Total RNA was extracted using the TRizol reagent (Life, USA). RNA was subjected to reverse transcription using RevertAid Reverse Transcriptase (Vazyme, Nanjing, China), followed by dilution of the resulting cDNA to a concentration of 100 ng/μL using RNase-free water. Real-time quantitative PCR (RT-qPCR) was performed on the CFX 96 Real-Time PCR system (Bio Rad, Hercules, CA, USA) using ChamQ SYBR qPCR Master Mix (Vazyme, Nanjing, China). The 20 μL RT-qPCR reaction system was consisted of 10 μL 2 × SYBR Premix Extaq, 1 μL of each primer (10 μM), 2 μL template (approximately 100 ng/μl) and 6 μl ddH_2_O. The procedure is as follows: denaturation at 95°C for 30 s (step 1), denaturation at 95°C for 5 s (step 2), primer annealing/extension and fluorescence signal collection at 60°C for 20 s (step 3). The next 40 cycles start in step 2. Three biological replicates were performed for each sample, and three technical repeats were performed for each replicate. Relative expression levels were determined with the 2^-ΔΔCt^ method (Livak and Schmittgen, 2001). ADP-ribosylation factor *Ta2291* whose expression was stable under various conditions, was used as an internal reference gene for RT-qPCR analysis. The primers shown in **Supplement File Table S3**.

### 2.11 Subcellular localization analysis of TaRSH2.7

To validate the subcellular localization of TaRSH2.7, the full length of TaRSH2.7 was amplified by 2 × Phanta Max Master Mix (Vazyme, Nanjing, China). The TaRSH2.7 sequences were recombined into 16318:TaRSH2.7:GFP vector with the restriction enzymes BamHI (NEB, Beijing, China) and ClonExpress II One Step Cloning Kit (Vazyme, Nanjing, China). Protoplasts were prepared from 30 healthy and tender wheat (Yangmai 20) leaves that grew for seven days. For the preparation method, refer to the wheat protoplast preparation and transformation kit (Coolaber, Beijing, China) (Liu et al., 2017). Observation with fluorescence microscopy (Nikon, Japan) 16 h after transformation.

## 3 Results

### 3.1 Identification and phylogenetic analysis of RSH members

**Figure 1:**
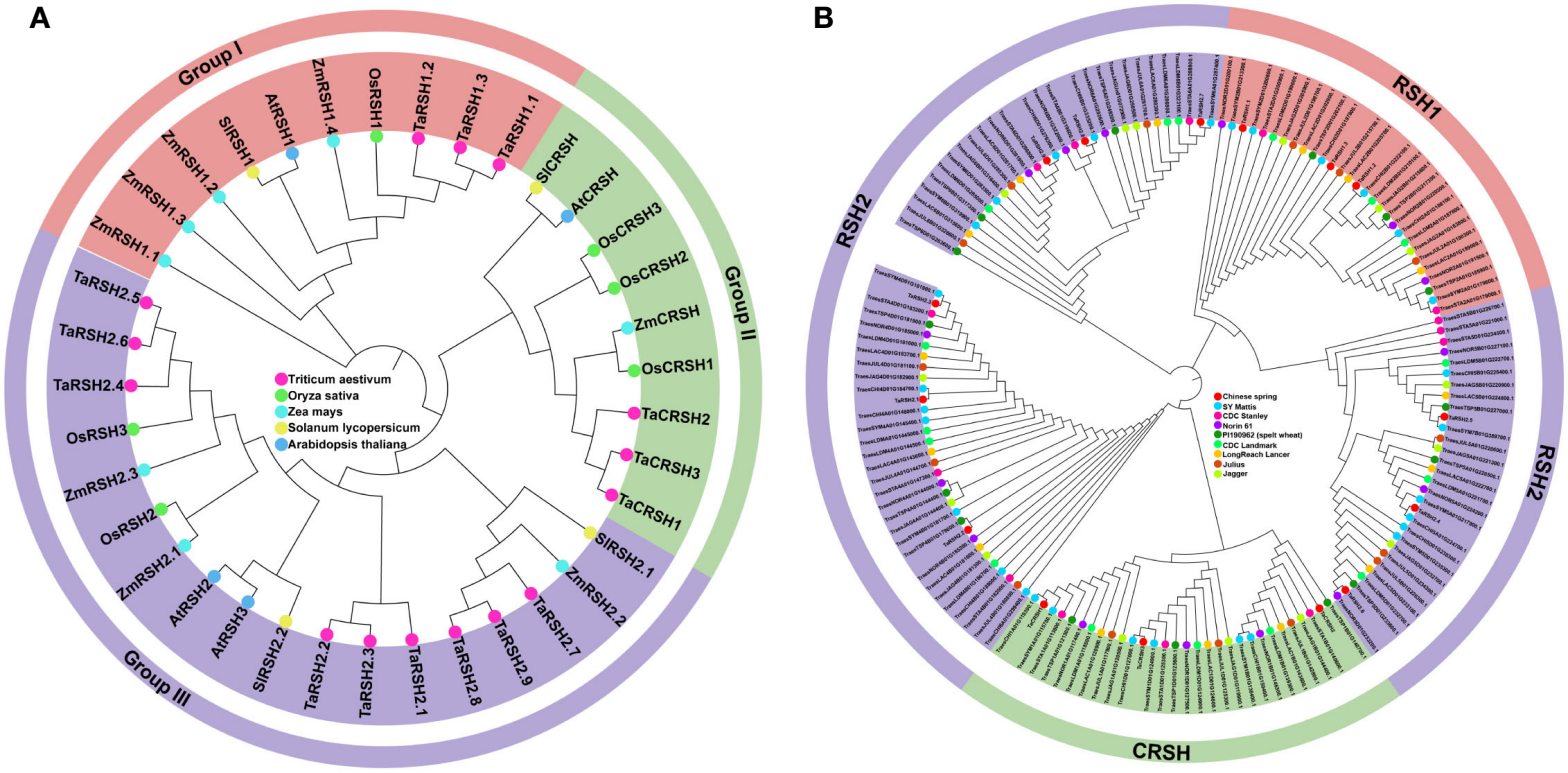
Phylogenetic analysis. **(A)** Phylogenetic analysis of *Triticum aestivum*, *Oryza sativa*, *Arabidopsis thaliana*, *Solanum lycopersicum* and *Zea mays* RSHs. **(B)** Phylogenetic analysis of Chinese spring, SY Mattis, CDC Stanley, CDC Landmark, Norin 61, PI190962 (spelt wheat), LongReach Lancer, Julius and Jagger RSHs.

**Figure 2:**
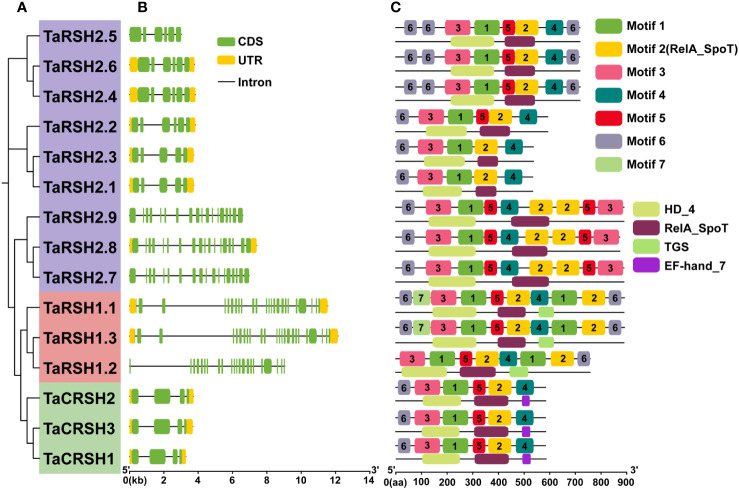
Exon–intron structure and conserved domain and motif analyses. **(A)** Phylogenetic tree of 15 TaRSHs; **(B)** Exon–intron analysis of 15 TaRSHs, the yellow and green box represent untranslated region (UTR) and coding sequence (CDS), and the black line represents intron. **(C)** Motif analysis of TaRSHs, Motifs were represented in different colors.

By employing a comprehensive bioinformatics approach, a total of 15 *RSH* genes were identified within the wheat genome. Aiming to study the evolutionary relationship of RSH genes of *T. aestivum*, *A. thaliana*, *O. sativa*, *S. lycopersicum* and *Z. mays*. a phylogenetic tree was constructed based on their amino acid sequence comparison. As illustrated in **Figure 1A**, based on the phylogenetic relationship and conserved domain, the 15 TaRSH proteins were named as TaRSH1.1 to TaRSH1.3, TaRSH2.1 to TaRSH2.6, and TaCRSH1 to TaCRSH3. The identified *TaRSH* genes were classified into three distinct groups: Group I contained three members, Group II contained three ones, and Group III contained nine members. In addition, all of the 15 TaRSHs possess RelA_SpoT and HD domain. Among them, TaRSH1.1, TaRSH1.2, and TaRSH1.3 belonging to Group I, contain TGS domain, which is a unique domain in Group I. TaCRSH1, TaCRSH2, and TaCRSH3 are members of Group II and possess the EF-Hand domain, which is a distinctive domain found exclusively in Group II (**Figure 2**). As shown in **Figure 1B**, phylogenetic analyses showed that the number and grouping of *RSH* genes in the cultivars were consistent with those of Chinese Spring, except for ArinaLrFor (No hits found), SY mattis (30) and CDC stanley (14, and lacked a RSH1).

### 3.2 Protein characterization and three-dimensional structure of TaRSHs

As shown in **Table 1**, protein characterization revealed that the amino acid number of TaRSHs with a distribution range of 534-899 aa, and the molecular weight with a distribution range of 58.85-98.91 kDa. The mean isoelectric point was pI of 6.78 with a distribution range of 6.03-7.62. The distribution range of instability index was 39.71-50.06, except for TaRSH1.2, other TaRSHs belonged to unstable proteins. The mean value of hydrophilicity was -0.26 and the distribution range was -0.39~-0.13. All the values of hydrophilicity were less than 0, These findings suggest that TaRSHs exhibit hydrophilic properties, indicating their tendency to interact with water molecules.

**Table 1:** The specific details of TaRSH protein characterization.

Name	Gene ID	Length^1^	MW^2^	pI^3^	Ins^4^	GRAVY^5^	Sub^6^
TaCRSH1	TraesCS1A03G0255000.1	582	64.18	6.74	44.55	-0.308	Cell membrane
TaCRSH2	TraesCS1B03G0355400.1	584	64.22	6.63	42.59	-0.258	Chloroplast
TaCRSH3	TraesCS1D03G0258800.1	583	64.21	6.61	44.41	-0.290	Cell membrane Chloroplast
TaRSH2.1	TraesCS2A03G0355800.1	534	58.85	7.06	49.27	-0.298	Cytoplasm Mitochondrion Nucleus
TaRSH2.2	TraesCS2B03G0475100.1	592	65.57	7.62	48.71	-0.318	Mitochondrion
TaRSH2.3	TraesCS2D03G0372500.1	536	59.12	7.30	50.47	-0.285	Chloroplast Mitochondrion
TaRSH1.1	TraesCS4A03G0311500.3	891	98.90	7.13	41.59	-0.223	Chloroplast
TaRSH1.2	TraesCS4B03G0469400.1	759	85.06	6.03	39.71	-0.205	Chloroplast
TaRSH1.3	TraesCS4D03G0420700.2	891	98.91	7.31	42.83	-0.229	Chloroplast
TaRSH2.4	TraesCS5A03G0543300.1	718	78.80	6.19	48.67	-0.374	Peroxisome
TaRSH2.5	TraesCS5B03G0552800.1	717	78.84	6.15	49.77	-0.369	Mitochondrion Peroxisome
TaRSH2.6	TraesCS5D03G0507200.1	716	78.79	6.28	50.06	-0.388	Mitochondrion Peroxisome
TaRSH2.7	TraesCS6A03G0717200.1	889	97.89	6.89	49.09	-0.132	Chloroplast
TaRSH2.8	TraesCS6B03G0851000.1	873	96.02	6.75	48.51	-0.153	Chloroplast
TaRSH2.9	TraesCS6D03G0600800.1	899	97.96	7.07	48.82	-0.137	Chloroplast

^1^(Amino acid length, aa); ^2^(Molecular weight, KD); ^3^(Isoelectric point); ^4^(Instability index); ^5^(Grand average of hydropathy); ^6^(predicted subcellular location.

Subcellular localization prediction analysis revealed that TaCRSH2, TaCRSH3, TaRSH2.2, TaRSH1.1, TaRSH1.2, TaRSH1.3, TaRSH2.7, TaRSH2.8, TaRSH2.9 were distributed in chloroplasts, while TaCRSH2 may be distributed in the cell membrane, and TaCRSH3, TaRSH2.4, TaRSH2.5 and TaRSH2.2 may be distributed in mitochondria; TaRSH2.4, TaRSH2.5, and TaRSH2.6 were distributed in peroxisome. In addition, TaCRSH1 is localized only in the cell membrane, while TaRSH2.1 is localized only in the nucleus.

**Figure 3:**
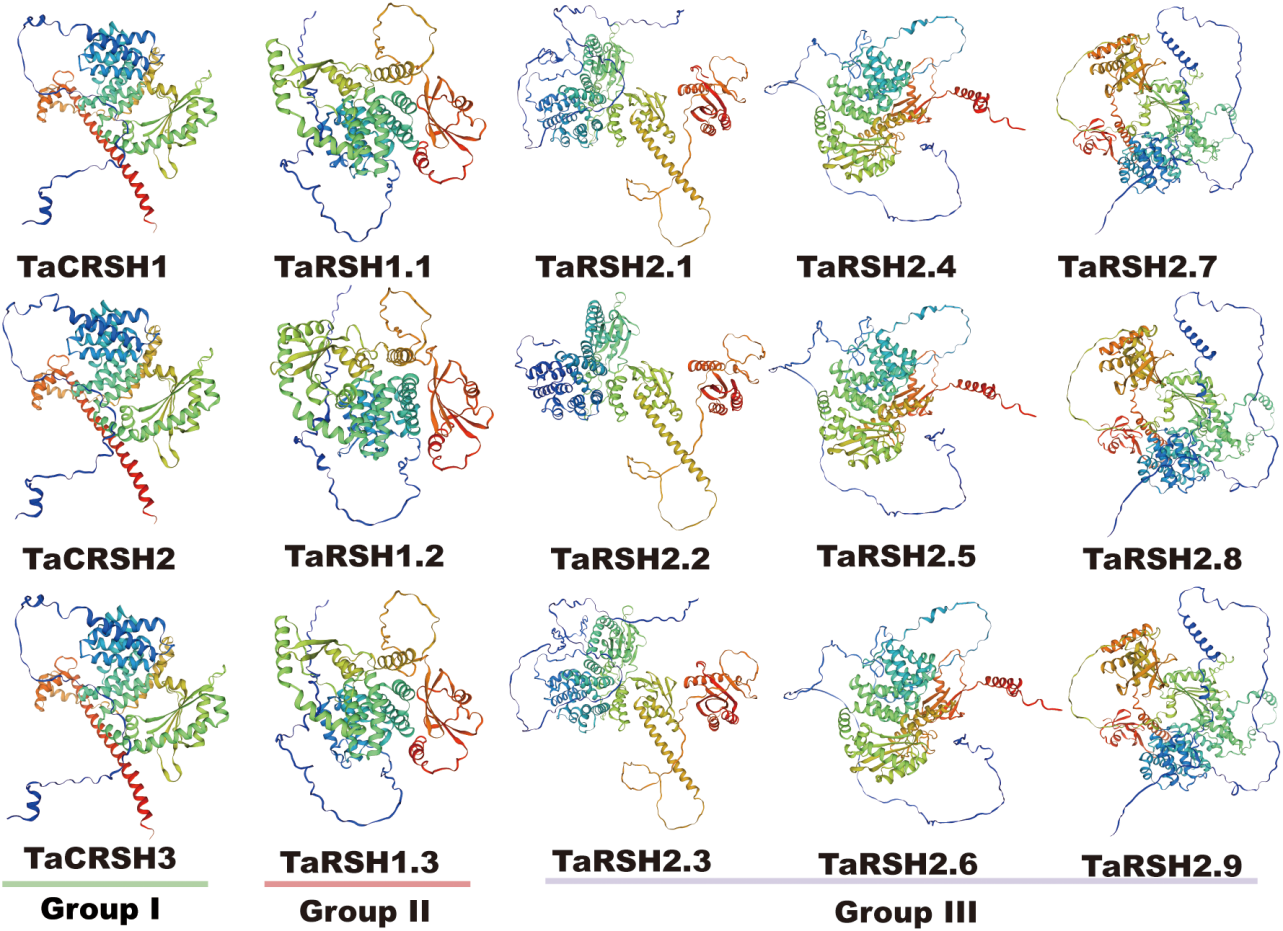
Predicted 3D models of TaRSHs. Models were generated by using SWISS-MODEL.

Three-dimensional homology modelling of TaRSHs was analyzed by SWISS-MODEL. As shown in **Figure 3**, all TaRSHs mainly consisted of *α*-helix, *β*-turn, random coil and extended strand. The proportion of these four structures in TaRSHs proteins varied, with *α*-helix accounting for the largest proportion (48.16%-58.43%), followed by Random coil (24.57%-37.33%), Extended strand (8.96%-16.64%), and the smallest proportion of *β*-turns (5.62%-9.76%). The Group II has the least amount of *α*-helix (47.55%), and the most amount of Extended strand (14.11%), *β*-turns (8.27%) and Random coil (30.08%).

### 3.3 Gene structure, motif and conserved domain analysis of TaRSHs

The structural diversity of exons and introns frequently influences the evolutionary trajectory of gene families, offering supplementary evidence that strengthens the phylogenetic clustering. (Shiu and Bleecker, 2003; Wang et al., 2014). In order to understand the structure characteristics of *TaRSHs*, the exon-intron structure of *TaRSH* genes was analyzed using GFF3 annotation of wheat. The gene structure of *TaRSH* gene has a relatively complex structure (**Figure 2**). Furthermore, the intron number of *TaRSH* ranging from 3-23. TaRSH1.3 contains 24 exons, while *TaCRSHs* only contains 3 exons of different lengths. The exon-intron structure shows that most *TaRSHs* have UTR non-coding regions at both ends of the sequence, and only *TaRSH2.5*, *TaRSH2.7*, *TaRSH2.9*, and *TaRSH1.2* have no UTR, indicating that the TaRSH family sequence is highly conserved.

Conservative motif analysis revealed that seven kinds of Motifs were identified from TaRSHs, among which TaRSH2.1 and TaRSH2.3 contained the least number of Motifs (four); TaRSH1.1 and TaRSH1.3 contained the most Motifs (10). By integrating phylogenetic analysis, it was observed that TaRSHs exhibiting a higher degree of relatedness also displayed a greater consistency in their motif composition. As shown in **Figure 2**, structural domain analysis revealed that Motif 2 contains the RelA_SpoT structural domain, which constitutes the key functional structural domain of RSH. There were additional motifs identified that did not exhibit a match with known key functional structural domains. The intricate and diverse structure of the *TaRSH* gene suggests its potential involvement in numerous crucial biological processes in plants.

### 3.4 Chromosomal mapping, collinearity and Ka/Ks analysis of *RSH* genes

**Figure 4:**
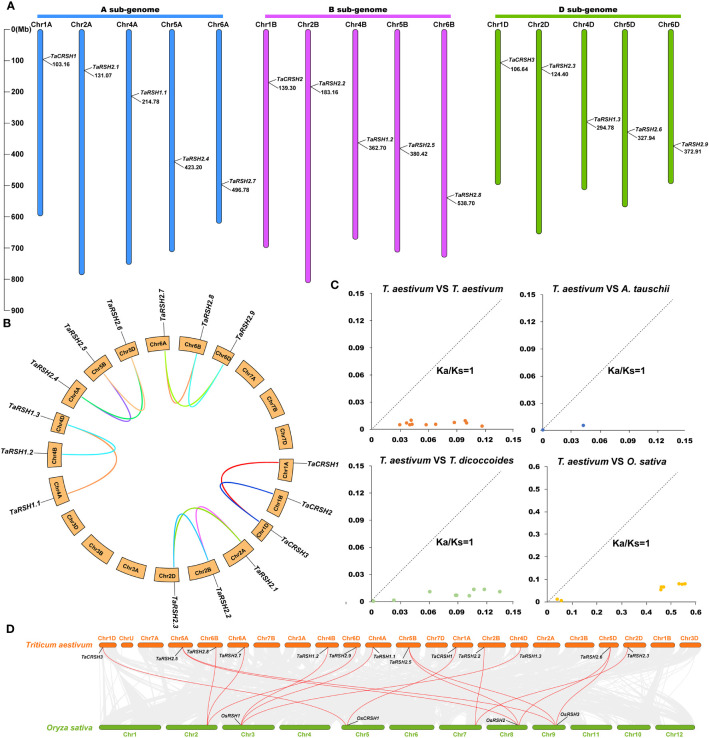
Chromosome location on wheat RSH gene and analysis of gene duplication. **(A)** Chromosomal location of 15 *TaRSH* genes in wheat genome. The lines represent gene duplication events. **(B)** TaRSHs collinearity analysis. **(C)** Ka/Ks ratio between RSH genes of Triticum aestivum, Triticum urartu, Triticum dicoccoides and Aegilops tauschii. **(D)** Collinearity analysis between wheat and between wheat and rice.

Based on the gene structure annotation information of TaRSHs, a chromosome distribution map was generated using MapInspect software. As exhibited in **Figure 4A**, 15 *TaRSHs* are evenly distributed on 15 chromosomes, and each sub-genome contains 5 *TaRSHs*. It is crucial of Tandem duplication and segmental duplication for the evolution of gene families (Liu et al., 2021). Through analysis of sequence similarity and chromosome positioning, it was observed that among the 15 *TaRSH* genes, a total of 13 pairs exhibited fragment duplications, whereas no tandem duplication events were detected. The findings indicate that the expansion of the RSH gene family in *T. aestivum* during evolution can primarily be attributed to fragment duplications (**Figure 4B**).

Through BLASTp analysis, 14, 12, and 8 *RSH* genes were identified from the reference genomes in *T. urartu, T. dicoccoides*, *and A. tauschii*. The Ka/Ks ratios for non-synonymous substitution rates (Ka) and synonymous substitution rates were calculated using TBtools. The results of Ka/Ks analysis revealed that all homologous gene pairs exhibited values below 1, indicating a notable presence of strong purifying selection pressure acting on the *RSH* gene within wheat species (**Figure 4C**). Based on the sequence similarity and chromosome position, a total of 13 pairs of fragment duplications were found among 15 *TaRSHs*, while no tandem duplication event was identified (**Figure 4D**).

### 3.5 *Cis*-acting element analysis of *TaRSHs*

**Figure 5:**
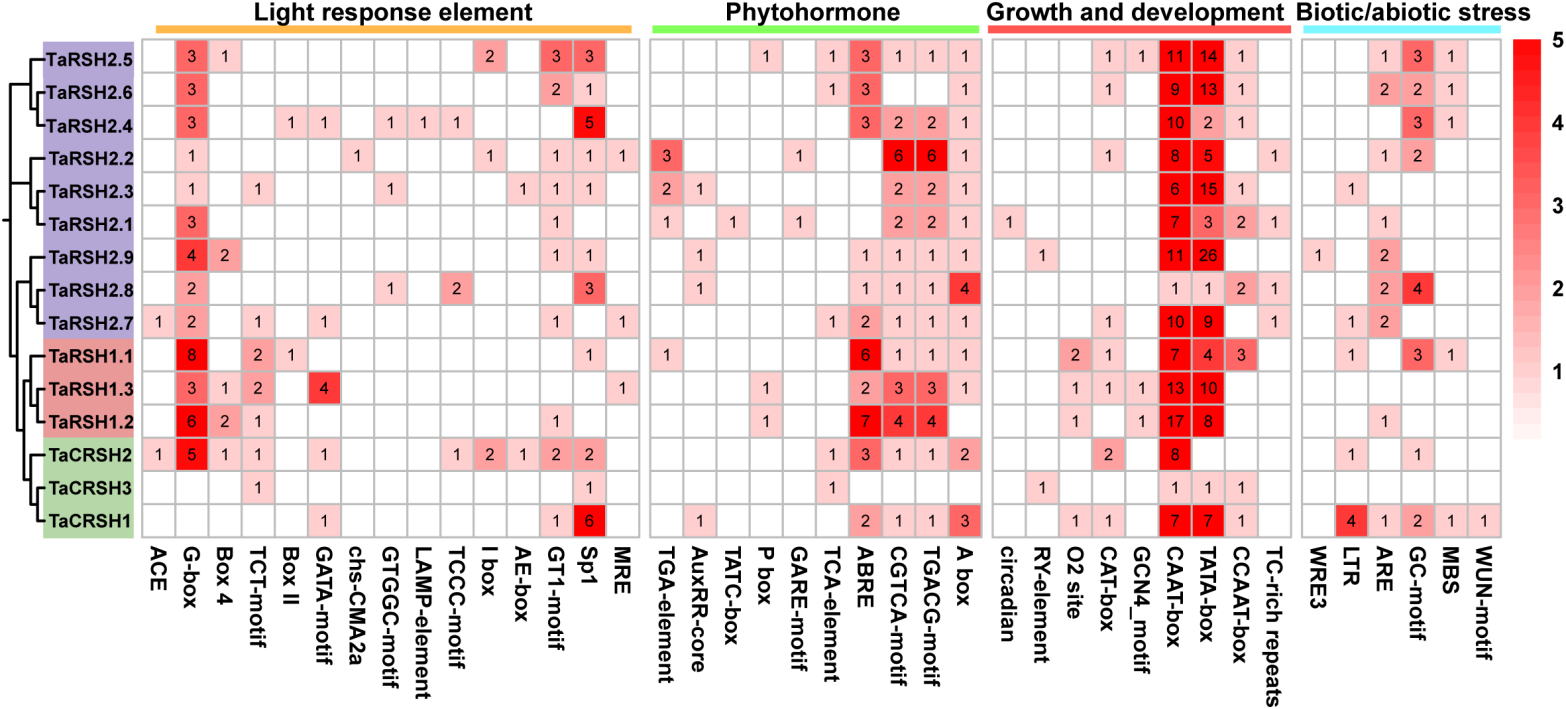
Statistical analysis of *cis*-acting elements in the promoter of TaRSH genes. The color and number of the grid represent the number of *cis*-acting elements in corresponding TaRSHs.

*Cis-*elements are one of the important factors in the modulation of gene expression. They are involved in regulating the expression of relevant genes for growth and developmental processes and adaptation to environmental changes (Liu et al., 2013). As indicated in **Figure 5**, 40 kinds of *cis*-elements were identified in total. In addition to a significant repertoire of fundamental components, such as TATA box and CAAT box, there are also many light response and hormone response elements. Among them, light responsive element includes 23 types, such as G-box, GA1 motif, Sp1, and MRE. Hormone response elements include regulatory gibberellin (GARE-motif, TATC-box and P-box), auxin (AuxRR-core and TGA-element), abscisic acid (ABRE), salicylic acid (TCA-element), and methyl jasmonate response element (TGACG-motif and CGTCA-motif), with abscisic acid and methyl jasmonate response element in the majority. Meanwhile, it also includes growth and development related *cis*-elements that circadian rhythm (circadian), meristem expression (CAT-box), and endosperm expression (GCN4_motif). In addition, anaerobic induction (ARE), anoxic specific inducibility (GC-motif), low-temperature (LTR), drought inducibility (MBS), wound responsive element (WUN-motif), and defense and stress responsiveness (TC-rich repeats) element also exist in the *TaRSHs* promoter region.

### 3.6 Protein interaction network analysis of TaRSHs

**Figure 6:**
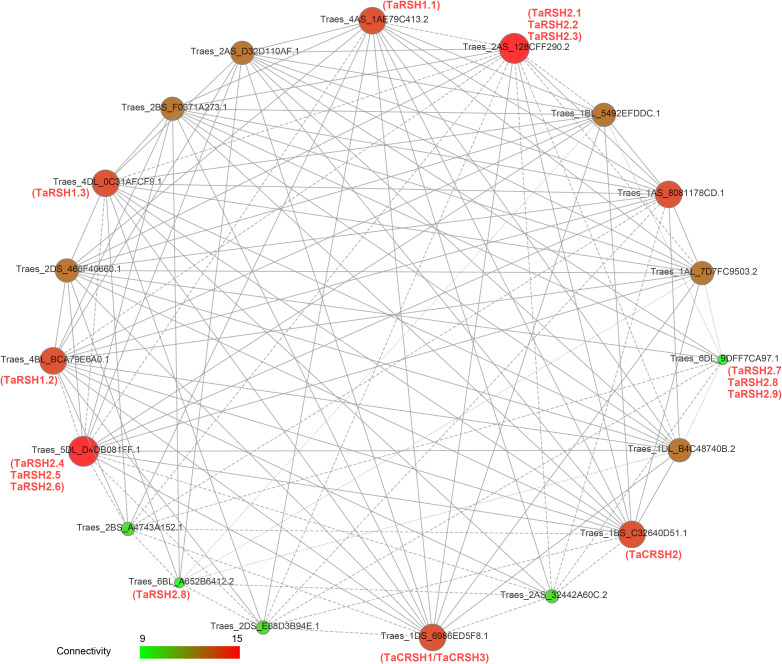
Network mapping between wheat RSH proteins. The interaction network between 15 TaRSH proteins and wheat-related transcription factors. The size of the ball and the intensity of the color both represent the intensity of the interaction. Green represents a small effect, and red represents a large effect.

Functional and physical interactions of TaRSH proteins were revealed with STRING software by association modelling. The protein interaction results (**Figure 6**) showed that TaRSH2.1, TaRSH2.2 and TaRSH2.3 may have potential interactions with Traes_2AS_128CFF290.2, while TaRSH2.4, TaRSH2.5 and TaRSH2.6 interact with Traes_5DL_D4DB081FF.1. TaRSH2.7, TaRSH2.8 and TaRSH2.9 have a low probability of interacting with Traes_6DL_9DFF7CA97.1. And the likelihood of TaRSH2.8 and Traes_6BL_A652B6412.2 interoperating is also low. We will follow up with yeast two-hybrid experiments to verify the accuracy of this result and explore their potential mutualistic networks even further.

### 3.7 Transcriptome analysis unveiled diverse expression patterns exhibited by *TaRSH* genes

**Figure 7:**
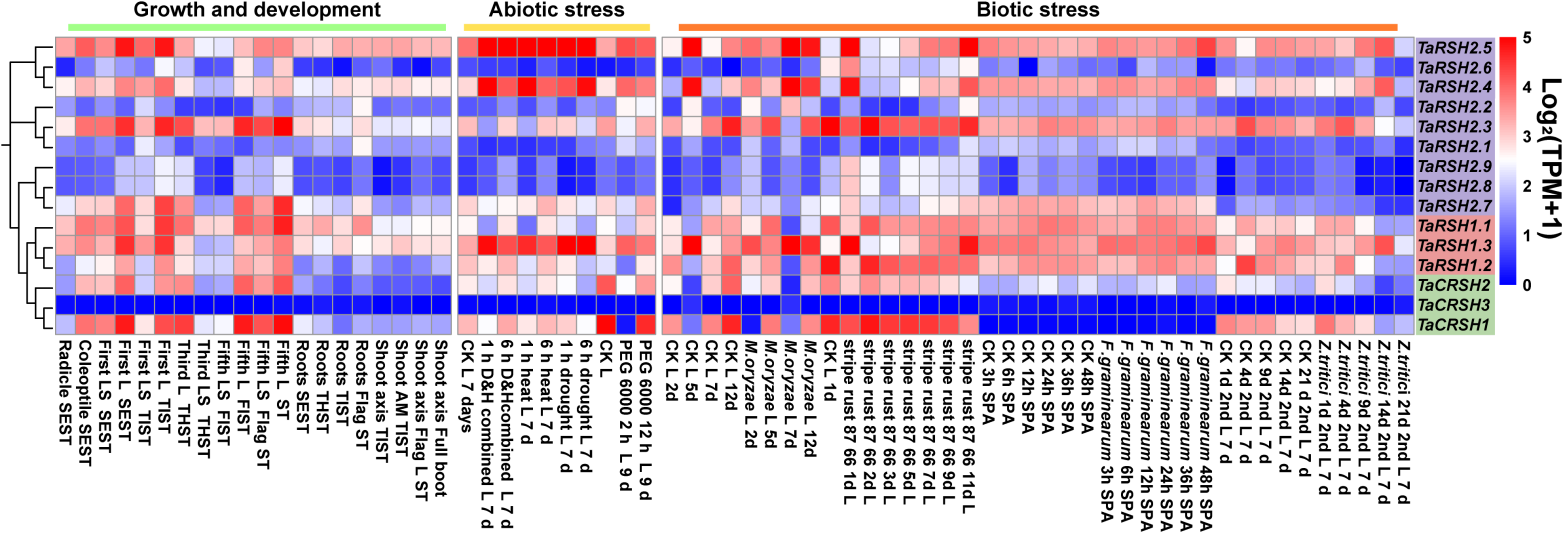
Expression pattern profiling of TaRSH genes under growth, development, and biotic/abiotic stresses. L, leaf; LS, leaf sheath; SEST, seeding Stage; TIST, tillering Stage; THST, three leaf Stage; FLST, fifth leaf Stage; SPA, spikelets anthesis; AM, apical meristem.

The analysis of expression patterns revealed that *TaRSH* genes exhibit diverse and distinct patterns of gene expression (**Figure 7**). During various growth and developmental stages, the expression levels of four *TaRSH* genes (*TaRSH1.3*, *TaRSH2.5*, *TaRSH2.4*, and *TaRSH2.3*) were significantly upregulated, whereas *TaCRSH1* were highly expressed only in the first leaf Seedling stage and the fifth leaf stage. In response to different biotic stresses such as *Zymoseptoria tritici*, *Magnaporthe oryzae*, stripe rust, and *Fusarium graminearum* infection, six *TaRSH* genes (*TaRSH1.1*, *TaRSH1.2*, *TaRSH1.3*, *TaRSH2.1*, *TaRSH2.2*, and *TaRSH2.3*) exhibited pronounced upregulation of their expression levels. In contrast, the expression levels of *TaCRSH1* were specifically upregulated only in response to stripe rust treatment. Under abiotic stress conditions, three *TaRSH* genes demonstrated significant induction, albeit at relatively lower expression levels individually. These findings imply the potential involvement of *TaRSH* genes in the defense response against abiotic stresses in wheat.

### 3.8 MicroRNA targeting analysis of *TaRSH* genes

**Figure 8:**
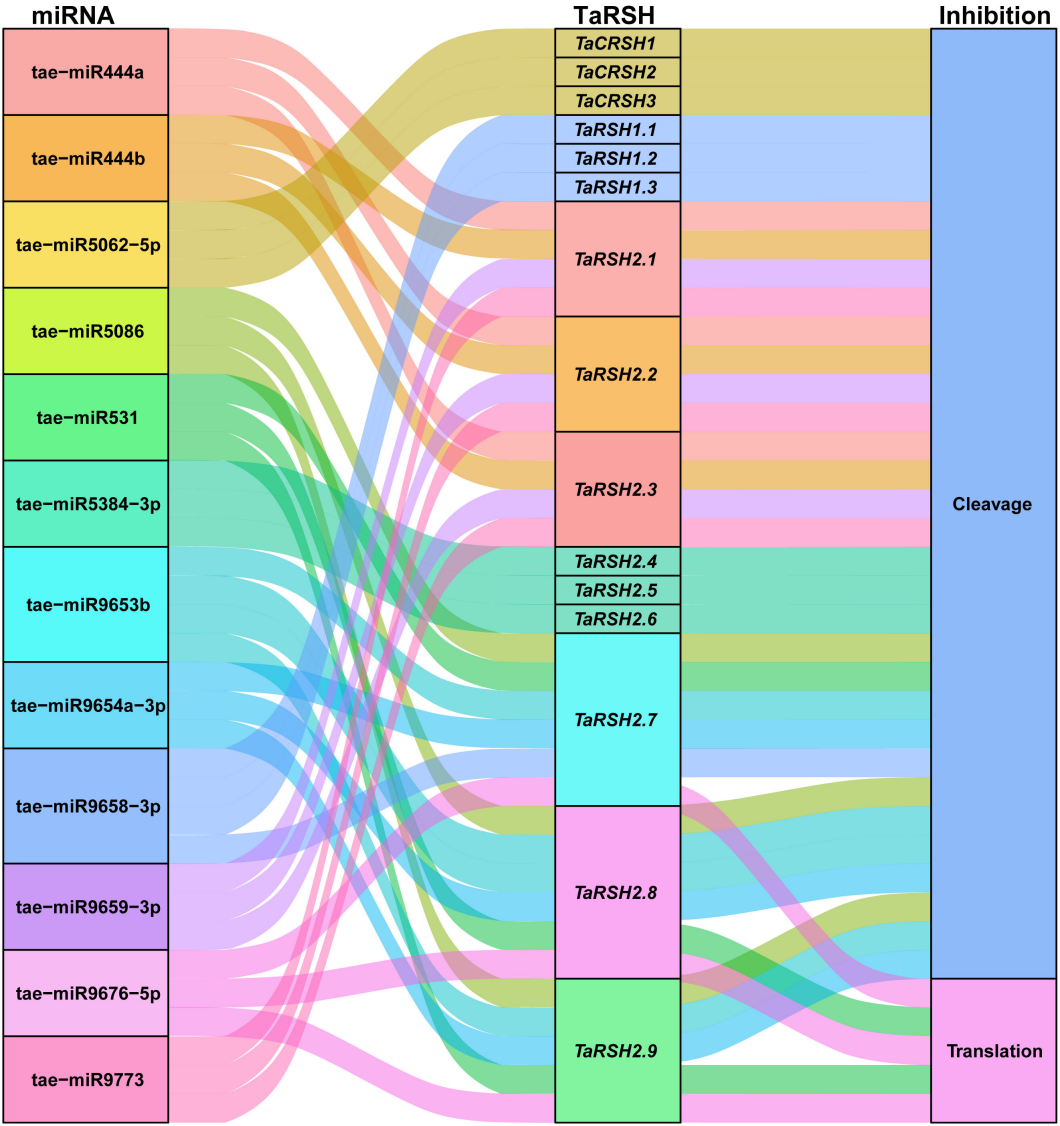
Sankey diagram for the targeting relationships between miRNA and ApCAT and corresponding inhibition pattern. The three columns represent miRNA, mRNA, and inhibition pattern.

MicroRNA (miRNA) have important functions in the modulation of gene expression. It can inhibit the degradation of the target gene mRNA or protein translation (Zhu et al., 2019). Consequently, we conducted a comprehensive analysis of potential miRNAs that could potentially target *TaRSH* genes, aiming to elucidate the regulatory mechanisms governing the expression of *TaRSH* genes and provide valuable insights into their regulation in wheat. As shown in **Figure 8**, 12 wheat miRNAs targeted 15 *TaRSH* genes by cleavage and translational repression, of which 10 miRNAs targeted 15 *TaRSH* genes by cleavage, and only tai-miR9676-5p targeted *TaRSH2.7*, *TaRSH2.8*, and *TaRSH2.9* by translational repression; and tai-miR531 targeted *TaRSH2.8* and *TaRSH2.9* by translational repression. It’s worth noting that tae-miR5062-5p only targets Group II members, and members in Group I are only targeted by tae-miR9658-3p, which imply tae-miR5062 and tae-miR9658 may have specificity and selectivity. The analysis showed that multiple miRNAs targeted *TaRSHs* and formed a miRNA-miRNA regulatory network to participate in the post-transcriptional expression regulation of *TaRSHs*.

### 3.9 RT-qPCR analysis of *TaRSH* genes

**Figure 9:**
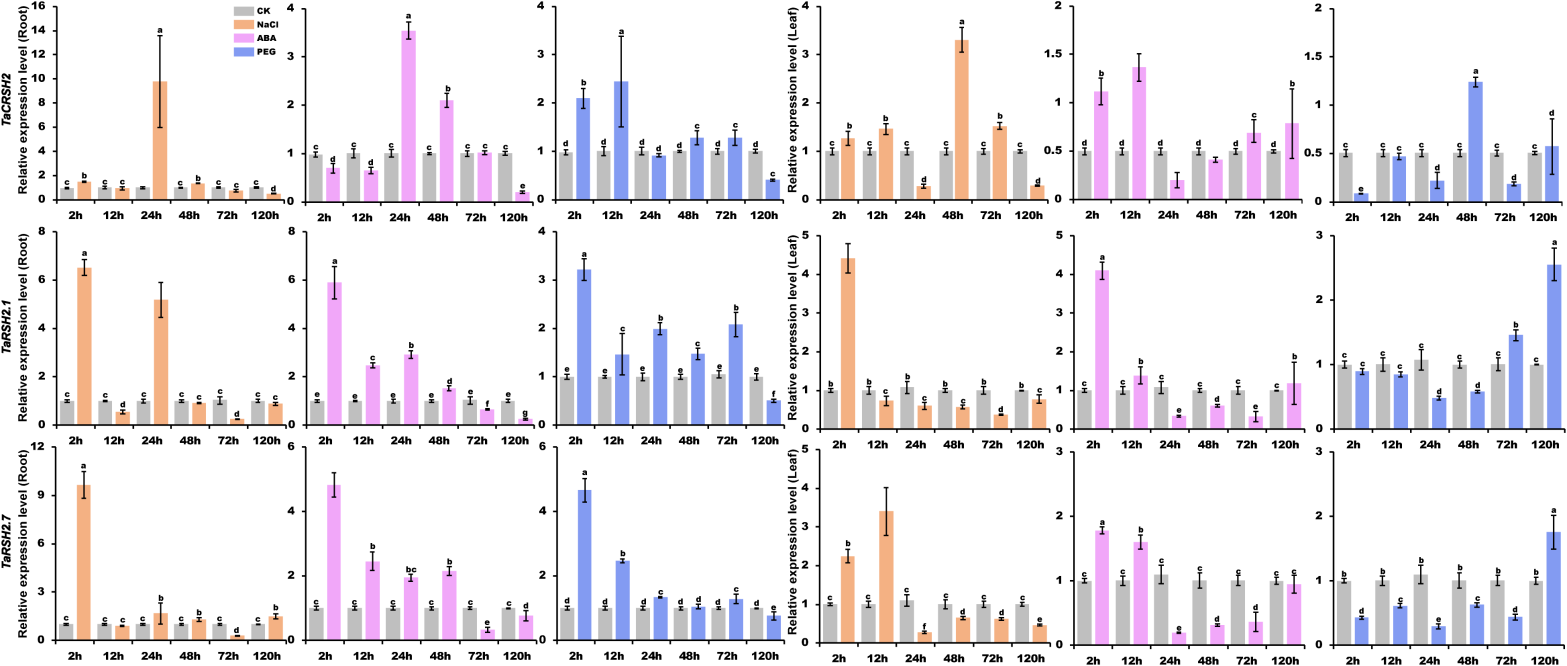
Analysis of TaRSH expression under abiotic stress. By using yangmai 20 for quantitative analysis, the treatments performed include 20% PEG, 150 mM NaCl and 100 μM ABA. Different colors represent different stress treatments. The Y axis represents the relative expression level, and the X axis represents the time point after stress treatment. Different letters in a column indicate significant differences between the treatments at p < 0.05 level.

To obtain a comprehensive comprehension of the functional significance of *TaRSH* genes in response to biotic and abiotic stresses, the expression patterns of three selected genes (*TaCRSH2*, *TaRSH2.1*, and *TaRSH2.7*) were analyzed using quantitative real-time PCR (RT-qPCR) in response to drought, ABA, and NaCl stresses (**Figure 9**).

In the presence of NaCl treatment, the expression patterns of *TaCRSH2* in both roots and leaves demonstrated notable divergence. In roots, the expression of *TaCRSH2* reached the maximum at 24 hours. Whereas in leaves, the maximum expression level was observed at 48 hours. Conversely, *TaRSH2.1* and *TaRSH2*.7 showed their highest expression levels at 24 hours in both roots and leaves, implying that *TaRSH2.1* and *TaRSH2*.7 exhibit a rapid response to salt stress in both above-ground and below-ground organs.

Under ABA treatment, the expression level of *TaCRSH2* showed a biphasic regulation pattern in roots, being initially down-regulated and subsequently up-regulated with the peak expression occurring at 24 hours. In contrast, in leaves, *TaCRSH2* was up-regulated followed by down-regulation, reaching its lowest expression level at 24 h. Remarkably, TaRSH2.1 and TaRSH2.7 demonstrated robust upregulation in both roots and leaves within 24 hours of ABA treatment, highlighting their rapid response to drought stress.

Under PEG treatment, the expression level of *TaRSH2.1*, *TaRSH2.7* and *TaCRSH2* was significantly upregulated at 2 hours in roots. In leaves, differentially expressed at 2 hours in leaves of *TaRSH2.7* and *TaCRSH2*, which implied that the two genes responded rapidly to drought stress and may play important regulatory roles in the response to adversity.

### 3.10 Subcellular localization of TaRSH2.7

**Figure 10:**
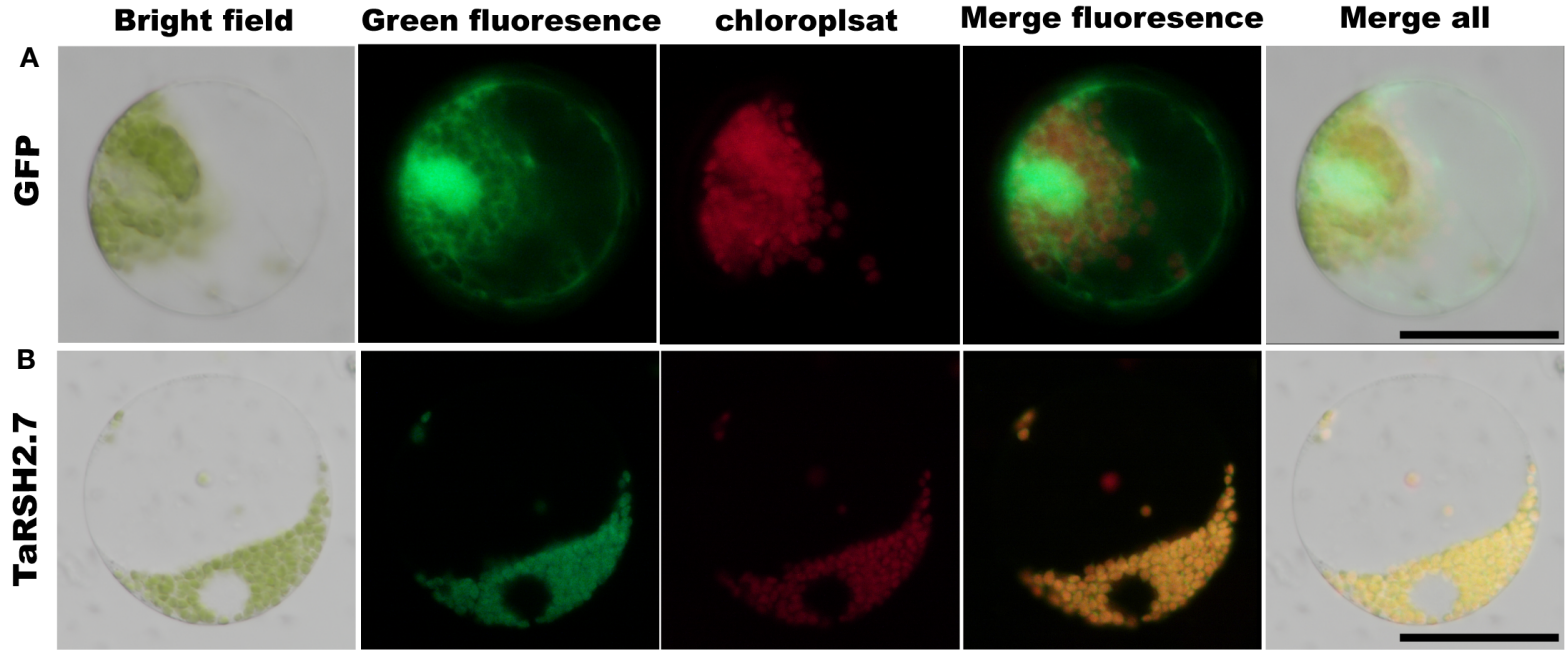
Subcellular location analysis of TaRSH2.7 was performed in wheat protoplast. The free GFP was as control **(A)**. TaRSH2.7 is localized on chloroplasts **(B)**. Green fluorescence represents free GFP and TaRSH2.7:GFP fusion protein, while red fluorescence indicates chloroplasts.

The predictions of subcellular localization showed that TaRSH2.7 is predicted localization in chloroplast. In order to validate the localization of TaRSH2.7 in the chloroplast, we employed a GFP tagging approach at the C-terminal of the protein and expressed it in wheat protoplasts. As a control, we also included free GFP (**Figure 10A**). Through fluorescence microscopy, we observed a distinct fluorescence signal specifically localized in the chloroplasts for TaRSH2.7:GFP, further confirming its chloroplast localization (**Figure 10B**). As a result, Consistent with the predicted results, TaRSH2.7 is localized in chloroplasts, suggesting its potential roles within this organelle.

## 4 Discussion

The (p)ppGpp not only plays a crucial role in microbial adaptation to adverse environments, but it also serves as a key regulator in plant growth, development, and stress response (Du et al., 2019). At present, genome-wide identification of RSH proteins has been performed in various plant species, including rice, *Arabidopsis*, and *B. napus* (Dąbrowska et al., 2021). Unfortunately, there is a lack of functional studies on wheat RSH, which significantly restricts our understanding of its biological roles and hinders the effective utilization of TaRSH in resistance breeding. Therefore, the systematic identification and analysis of wheat *RSH* gene at the genome level and understanding its expression pattern in drought and salt stresses, can lay the foundation for studying the stress response function of *TaRSH*.

In this study, 15 *TaRSH* family members were identified from the wheat genome, which encoded proteins have a length of 534-899 aa (**Table 1**), and their protein lengths are similar to those of AtRSHs (558-883 aa) (van der Biezen et al., 2000) and OsRSHs (559-892 aa) (Ito et al., 2022). Meanwhile, combined with phylogenetic relationships and conservative domain analysis, it was found that OsRSHs and TaRSHs were located in the same subgroup exhibiting significant structural similarity. For example, OsRSHs have EF-hand structural domains and are only specifically present in CRSH, which may be involved in Ca^2+^ metabolism or play a crucial role in Ca^2+^ metabolism, and TaCRSHs also contain EF-hand structural domains. So we speculate that TaCRSHs may be involved in Ca^2+^ metabolism as well. In addition, OsRSH1 contains a conserved TGS functional domain, which may have a (p)ppGpp hydrolysis function, and TaRSH1 also contains a TGS structural domain, so we speculate that TaRSH1 may also be involved in the hydrolysis of (p)ppGpp (Du et al., 2019).

Segmental duplication and tandem duplication are two primary mechanisms underlying gene copy number variations, subsequently leading to the expansion of plant gene families (Huang et al., 2002). In this study, we identified 13 pairs of segmental duplicated gene pairs based on chromosomal location and collinearity analysis, while no tandem duplication events were detected. Therefore, segmental duplication is considered as the primary mechanism driving the expansion of the wheat *RSH* gene family during the evolutionary process (Li et al., 2022). The Ka/Ks value, also known as the ratio of non-synonymous to synonymous substitution rates, serves as a quantitative indicator for assessing the rate of genetic evolution (Hurst, 2002). The Ka/Ks value of 1 suggests neutral selection, while a Ka/Ks value higher than 1 indicates positive selection leading to accelerated evolution. Conversely, a Ka/Ks value lower than 1 suggests purifying selection due to functional constraints (Kawaura et al., 2009). To further investigate selective pressures, we calculated the Ka/Ks values for inter-specific wheat and wheat with three ancestral wheat homologous gene pairs. The result showed that Ka/Ks values of all homologous gene pairs were all less than 1. This result suggests that *RSH* is highly conserved not only in wheat, but also among ancestral wheat.

The *cis*-regulatory elements in the promoter region are the major factors regulating gene expression, which play a crucial role in regulating the expression of target genes in response to developmental processes and environmental changes (Yin et al., 2020). Previous studies have shown that *OsRSHs* contains a large number of light-responsive elements, and its expression is regulated by light (Du et al., 2019). Similarly, in this study, we observed that *TaRSHs* contain the highest number of light-responsive elements, suggesting a potential correlation between the expression of *TaRSHs* and light conditions. Meanwhile, a variety of elements related to biotic/abiotic stress response were also detected, which indicate that *TaRSHs* play a role in the stress response. In addition, plant hormone response elements such as abscisic acid (ABRE), jasmonic acid (CGTCA-motif and TGACG-motif), gibberellin (TATC-box) and salicylic acid (TCA-element) were also found in the *TaRSHs* promoter. Related studies have shown that these plant hormones play an important role in plant response to stress. For example, TaIAA15-1A can enhance drought tolerance in *Brachypodium* by regulating abscisic acid signal pathway (Su et al., 2023). Ellen H Colebrook study shows that plants can flexibly adjust the level of gibberellin in response to a variety of stresses, such as salt, drought, cold, shading and submergence (Colebrook et al., 2014). These results further imply that *TaRSHs* have a crucial function in plant response to multiple stresses.

To further reveal the participating roles of *TaRSHs* in different growth and development stages and biotic/abiotic stresses, its expression pattern profiling was performed by mining massive RNA-seq data. The results showed that the expression level of *TaRSHs* in leaves was much higher than that in roots and stems, suggesting *TaRSHs* expression was tissue-specific. This result is consistent with the conclusion that RSH plays a biological role in chloroplasts (Boniecka et al., 2017). Meanwhile, Du’s study show that during the initial stages of high-temperature treatment, the relative expression levels of *OsRSH1* are significantly reduced, while *OsRSH2* is significantly induced with expression levels gradually increasing within 6 hours (Du et al., 2019). Similarly, multiple *TaRSHs*, such as *TaCRSH2*, *TaRSH1*.*1*, and *TaRSH2.3*, were significantly upregulated under high temperature stress within 6 hours. In addition, the expression level of *TaRSHs* is also up-regulated to varying degrees by fungal pathogens such as *F. graminearum*, *Z. tritici*, and *M. oryzae*. The above results showed that *TaRSHs* not only participates in the growth and development of wheat, but also plays a positive regulatory role in stress response processes.

To explore the biological function of *TaRSHs* and reveal its expression characteristics in response to abiotic stress, in this study, based on the transcriptomic expression profiling results, three differential expression genes (*TaCRSH2*, *TaRSH2.1*, and *TaRSH2.7*) were selected for further analyzing their expression characteristics under salt and drought. The results showed that the results indicate that under drought, ABA, and salt stress, *TaCRSH2*, *TaRSH2.1*, and *TaRSH2.7* in both leaf and root tissues are induced and upregulated to varying degrees at specific time points, which suggest *TaRSHs* can participate in the response process of wheat to stresses including drought, ABA, and salt. Previous study had shown that *OsRSHs* (Du et al., 2019) and *AtRSHs* (Hirayama and Shinozaki, 2007; Mizusawa et al., 2008) also can be induced by drought, salt, and ABA, which is consistent with our research results.

## 5 Conclusions

In this study, a total of 15 *RSH* genes were discovered within the wheat genome. These genes were categorized into three distinct groups based on their conserved domains and evolutionary relationships. Interestingly, the 15 *TaRSHs* were found to be evenly distributed among the 15 chromosomes of wheat. Furthermore, the *RSH* gene exhibited a high degree of conservation not only within wheat species but also across its three ancestral wheat species. It was evident that RSH has experienced significant selective pressure, leading to substantial purification during its evolutionary process. Protein characterization showed that TaRSH proteins are mostly hydrophilic unstable acidic protein. The analysis of *cis*-elements and transcriptome show that TaRSH plays a significant role in plant growth, stress response and development. The results of RT-qPCR analysis showed that *TaCRSH2*, *TaRSH2.1* and *TaRSH2.7* participated in the process of wheat response to drought, ABA and NaCl stresses. These studies show that the *RSH* gene family of wheat is closely related to plant growth and development, stress response and plant hormones. At the same time, it is necessary to identify and study functional genes to provide useful information for further exploring the biological functions of the wheat *RSH* gene family.

## Data availability statement

The original contributions presented in the study are included in the article/**Supplementary Material**. Further inquiries can be directed to the corresponding authors.

## Author contributions

MW: Writing – original draft, Writing – review & editing. WH: Writing – review & editing. YW: Writing – original draft. XH: Data curation, Formal Analysis, Software, Writing – review & editing. WC: Data curation, Investigation, Writing – original draft. SW: Funding acquisition, Project administration, Writing – original draft. YZ: Methodology, Writing – original draft. WW: Funding acquisition, Writing – review & editing.
